# Cognitive decline in Sprague–Dawley rats induced by neuroplasticity changes after occlusal support loss

**DOI:** 10.1111/cns.14750

**Published:** 2024-06-19

**Authors:** Xiaoyu Wang, Qian Pang, Jiangqi Hu, Bin Luo, Yunping Lu, Xu Sun, Shixiang Meng, Qingsong Jiang

**Affiliations:** ^1^ School of Stomatology Capital Medical University Beijing China; ^2^ Department of Prosthodontics, Beijing Stomatological Hospital Capital Medical University Beijing China

**Keywords:** cognitive impairment, hippocampus, neuroplasticity, tooth loss

## Abstract

**Background:**

Tooth loss is closely related to cognitive impairment, especially affecting cognitive functions involving hippocampus. The most well‐known function of the hippocampus is learning and memory, and the mechanism behind is neuroplasticity, which strongly depends on the level of brain‐derived neurotrophic factor (BDNF). While research has delved into the possible mechanisms behind the loss of teeth leading to cognitive dysfunction, there are few studies on the plasticity of sensory neural pathway after tooth loss, and the changes in related indicators of synaptic plasticity still need to be further explored.

**Methods:**

In this study, the bilateral maxillary molars were extracted in Sprague–Dawley rats of two age ranges (young and middle age) to establish occlusal support loss model; then, the spatial cognition was tested by Morris Water Maze (MWM). Quantitative real‐time PCR (qPCR) and Western Blotting (WB) were used to detect BDNF, AKT, and functional proteins (viz., PSD95 and NMDAR) of hippocampal synapses. Golgi staining was used to observe changes in ascending nerve pathway. IF was used to confirm the location of BDNF and AKT expressed in hippocampus.

**Results:**

MWM showed that the spatial cognitive level of rats dropped after occlusal support loss. qPCR, WB, and IF suggested that the BDNF/AKT pathway was down‐regulated in the hippocampus. Golgi staining showed the neurons of ascending sensory pathway decreased in numbers.

**Conclusion:**

Occlusal support loss caused plastic changes in ascending nerve pathway and induced cognitive impairment in rats by down‐regulating BDNF and synaptic plasticity.

## Graphical abstract

The model of occlusal support loss in rat was established in order to verify the adaptive changes of ascending sensory pathway, including periodontal tissue, trigeminal ganglia, and hippocampus, detected by pathological methods. Synaptic plasticity related AKT pathway and the expression of downstream functional proteins were detected. The results confirm that ascending pathway had plastic changes in structure, and synaptic plasticity related factors in hippocampus was down‐regulated, resulting in decreased spatial cognitive function.

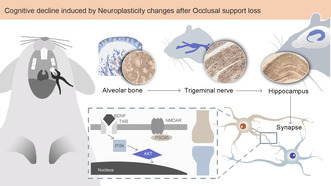

## 1 INTRODUCTION

Many studies reported that tooth loss can lead to cognitive impairment, especially in memory loss.^1^, ^2^, ^3^ Despite possible pathways such as mastication reduction pathway,^4^ enhanced neurodegeneration pathway,^5^ and chronic inflammation pathway^6^ have been proposed, however, the exact mechanism remains unclear. Oral‐maxillofacial system is strongly connected to the cerebral system, developmentally and anatomically. The actions of sucking and chewing are believed to contribute to the early development of nerves. During mastication, peripheral receptors in dental pulp and periodontal membrane are activated.^7^ Feelings (e.g., pain and proprioception) can be transmitted to the trigeminal nerve and mainly arrived at the first and second primary somatosensory representative areas of the face (SI/SII).^8^ Cognition‐related regions such as the hippocampus, prefrontal cortex, entorhinal cortex were also found to be activated during this process.^9^, ^10^

Memorize is a pivotal cognitive function. The molecular mechanism of memory has been found to be neuroplasticity.^11^, ^12^ Neuroplasticity includes macroscopic changes such as brain activation variation and gray matter/white matter volume changes.^13^, ^14^ Microscopic change happens in the synaptic level, with includes structural and functional variations.^15^, ^16^ Synaptic plasticity is related to a variety of neurotrophic factors, among which brain‐derived neurotrophic factor (BDNF) has been proved to be one of the key mediators.^17^ BDNF is a well‐known upstream factor that activates a variety of pathways,^18^ among which, AKT signaling pathway plays a fundamental role in maintaining cell proliferation and inhibiting cell apoptosis.^19^, ^20^ BDNF could also influence synaptic functions by regulating the distribution and quantity of functional proteins, such as N‐methyl‐D‐aspartate receptor (NMDAR) and Postsynaptic density protein 95 (PSD95), which participate in forming postsynaptic density (PSD), a fundamental structure of synapses.^21^, ^22^, ^23^

Previous studies have shown that loss of teeth can lead to decreased activation and degenerative changes in hippocampus.^24^, ^25^, ^26^, ^27^ As occlusal  support loss must be accompanied by reduction and loss of masticatory activation, we assume that not only the ascending nerve pathway, but also the hippocampus will show negative plasticity changes, which may explain the memory loss. However, there are few studies on the plasticity of sensory neural pathway between periodontal tissue and hippocampus after tooth loss, and the role of BDNF and synaptic plasticity in this process still needs to be further explored.

## 2 METHODS

### 2.1 Animals

Animals were used according to the animal care guidelines established by Capital Medical University. All methods are reported in accordance with ARRIVE 2.0 guidelines for the reporting of animal experiments. 18 male Sprague–Dawley rats aged 5 weeks and 18 aged 8 months were randomly divided into young tooth loss (YL) group, young control (YC) group, middle‐aged tooth loss (OL) group, and middle‐aged control (OC) group (*n* = 9 each group). The animals were housed under standard conditions and exposed to a 12‐h light–dark cycle and had an unlimited access to food and water.

### 2.2 Tooth‐extraction operation

All rats were anesthetized by intravenous administration of pentobarbital sodium (15‐40 mg/kg). Anesthesia was performed in all groups, and all maxillary molars were extracted in YL and OL groups. It was necessary to remove any dental root that might remained. After the operation, the rats were placed on cotton pad and under warm light. The activity of rats was observed closely 1 week after operation. Body weight was recorded since operation. The behavioral test was conducted after the body weight recovered to the preoperative level and increased steadily for more than 2 weeks.

### 2.3 Morris Water Maze test

It took 1 week for the rats to adapt the environment. A circular pool with a diameter of 1.3 m was used. The water depth is 0.6 m and temperature is constant at 24 ± 2°C. Markers divided the pool into four quadrants. The whole experiment was recorded by camera. The experiment lasted for 5 days, and one platform was strictly fixed except for the last day. During the navigation test stage (1–4 days), each rat received directional navigation training 4 times a day, after entering water for 90s, rats should climbed up the platform independently or being guided artificially for a 10 second stay. On the last day, the platform was removed and each rat was given two spatial probe tests. The time from the rat dropping into water to a first crossing platform area was recorded as the time of first passing platform (TFPP). Within 90 seconds, the rats have been observed and the number of crossing the platform (FPP) was recorded either.

### 2.4 Real‐time polymerase chain reaction

Hippocampus was isolated to extract total RNA (RNA simple Total RNA Kit, TINAGEN®, China) and converted into cDNA (FastKing RT Kit With gDNase, TINAGEN®, China). SYBR Green chimeric fluorescence method was used for real‐time PCR (SuperReal PreMix Plus SYBR Green, TIANGEN®, CHINA/ABI 7500 real‐time quantitative PCR instrument, ThermoFisher Scientific, USA). The primer designs were as follows: Rat Brain‐derived neurotrophic factor (*Bdnf*) is 5‐CCATAAGGACGCGGACTTGT‐3 and 5‐GAGGCTCCAAAGGCACTTGA‐3; Rat Protein kinase B‐1(*Akt1*) is 5‐GTGGCAAGATGTGTATGAG‐3 and 5‐CTGGCTGAGTAGGAGAAC‐3; Rat N‐methyl‐D‐aspartate receptor 2B (*Nr2b*) is 5‐CGCATCTGTCCACCATT‐3 and 5‐GCATCAGGAAAGCCTCG‐3.

### 2.5 Western blot analysis

Hippocampus was isolated to extract total protein, and the concentration was calculated based on the results obtained from the Bradford method analysis. After dilution, the samples were implemented at the same volume (4%–15% MP TGX Gel 10 W 30 μL, pkg 10). 80 V to 120 V electrophoresis (Bio‐Rad, PowerPac™ base electrophoresis) for about 40 min, and the Bio‐Rad Trans‐Blot Turbo for 7 min. They were quickly placed in TBST at room temperature and washed in a shaker. After that, non‐specific sites were sealed with 5% skim milk for 2 h. The primary antibodies such as BDNF (1/ 1000; ab108319, Abcam), AkT1/2/3 (1/10,000; ab179463, Abcam), NMDAR (1/1000; ab254356, Abcam), PSD95 (1/1000; ab18258, Abcam), and GAPDH (1/10,000; ab8245, Abcam) were incubated overnight in a shaking bed at 4°C. The secondary antibody was incubated at room temperature for 1 h and was fully developed in a Bio‐rad ChemiDoc Touch Imaging System using a developer solution (hypersensitive ECL Chemiluminescence kit, NcmECL Ultra).

### 2.6 Golgi staining

The maxillary alveolar bone, trigeminal nerve, and brain of rats were fixed with paraformaldehyde at 4°C for 48 h. The nerve tissue and decalcified bone (Plank‐Rychlo decalcification solution, Shang Po Biotechnology Co., LTD., Shanghai) were stained with modified Golgi silver plating (nerve tissue slice dyeing Kit, Bioesn, Shanghai). The solution was dyed by medium A for 2–3 days at 37°C. Wash with distilled water for 1–2 min and dye with medium B for 2–3 days at 37°C. The tissue blocks were dehydrated by ethanol and embedded in paraffin. The thickness was 20 μm. Optical microscope was used for observation. The number of silver stained neurons was analyzed by Image‐Pro Plus 6.0.

### 2.7 Immunofluorescence

The brain was fixed and dehydrated and cut into coronal frozen sections with a thickness of 30 μm (Leica CM3050 S). BDNF (0.33–20 μg/mL; ab205067, Abcam), AkT1/2/3 (1/10,000; ab179463, Abcam), and Nissl staining was incubated overnight with 4°C shaker. Goat Anti‐mouse IgG H&L (Alexa Fluor® 647) and Goat anti‐rabbit IgG H&L (Alexa Fluor® 488) were used as secondary antibodies. After incubation, images were collected by laser confocal microscopy (Leika, TCS SP5). Average fluorescence intensity was analyzed by Image‐Pro Plus 6.0.

### 2.8 Statistical analyses

SPSS 23.0 (IBM, Armonk, NY, USA) was used for data analysis. Data were tested by Kolmogorov–Smirnov for normal distribution. Data that conform to the normal distribution are represented as mean ± standard deviation. The experimental data of each group were normally distributed, conforming to the homogeneity of variance test. The t test was used for one‐way analysis of variance. *p* < 0.05 was considered to be statistically significant.

## 3 RESULTS

### 3.1 Changes in body weight of rats after tooth extraction

**FIGURE 1:**
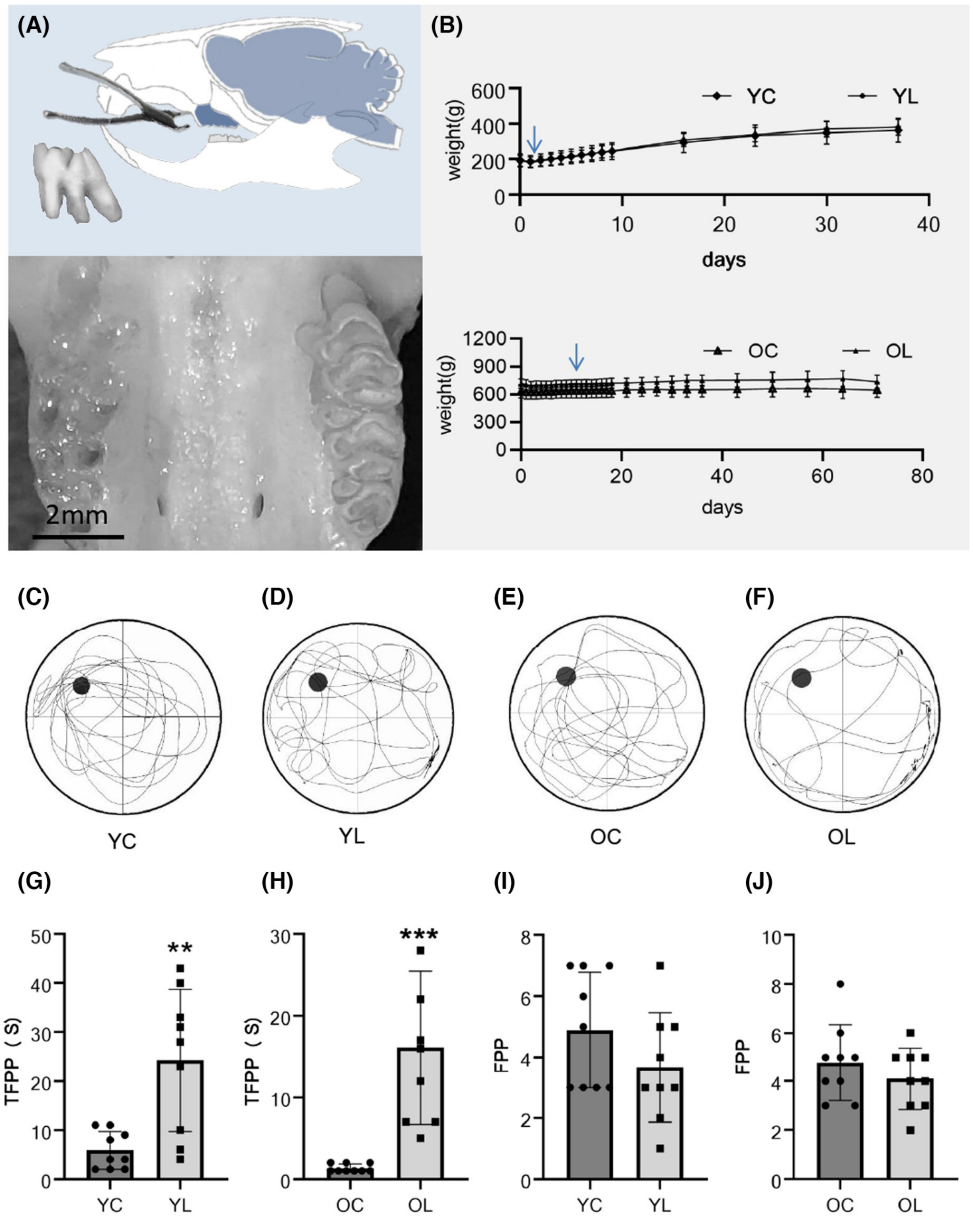
Trend in weight change and results of MWM. (A) Maxillary molars were extracted without root breakage. (B) The body weight of rats in the YL and OL groups returned to preoperative levels as indicated by the arrow within one and 2 weeks respectively. The body weight increased steadily in the following days. (C–F) Swimming tracks were recorded in each group, rats in YC and OC groups tended to explored in circles around the platform area, while rats in the YL and OL groups were swimming randomly in the pool. (G) The average time taken for the first time passing the platform (TFPP) in the YL group was significantly longer than that for the YC group (*p* < 0.01). (H) The average time taken for the TFPP in the OL group was significantly longer than that for the OC group (*p* < 0.001). (I, J) The frequency of passing the platform area (FPP) was less in the YL and OL groups than in the YC and OC groups. However, the difference was not significant (*p* > 0.05). Values were means ± SEM of determinations with *n* = 9 for each group. *p < 0.05, **p < 0.01, ***p < 0.001.

The extraction socket was clean without remained dental roots (Figure 1A). The weight of YL group recovered to the preoperative level in 2 days and increased continuously. The weight gaining and observing period lasted for 37 days, when the MWM test was conducted. The body weight of OL group recovered to the preoperative level 13 days after tooth extraction, and the behavioral experiment was conducted on day 72 (Figure 1B).

### 3.2 Worse behavioral performance after tooth extraction

Swimming routes of rats were recorded (Figure 1C–F). TFPP of YL group was 25.6 ± 13.6 s, and YC group was 5.9 ± 3.9 s. TFPP of YL group was longer than YC group (*p* < 0.01) (Figure 1G). TFPP of OL group was 16.2 ± 8.8 s, and OC group was 1.3 ± 0.5 s. TFPP of OL group was significantly longer than OC group (*p* < 0.001) (Figure 1H). FPP in YL group was 3.7 ± 1.8 times, and YC group was 4.9 ± 1.9 times. FPP in YL group was less than YC group, but there was no statistical difference (*p* > 0.05) (Figure 1I). FPP in OL group was 4.1 ± 1.3 times, and OC group was 4.8 ± 1.5 times. FPP of OL group was less than OC group, but there was no statistical difference (*p* > 0.05) (Figure 1J).

### 3.3 Decreased level of gene expression in synaptic‐plastic factors

**FIGURE 2:**
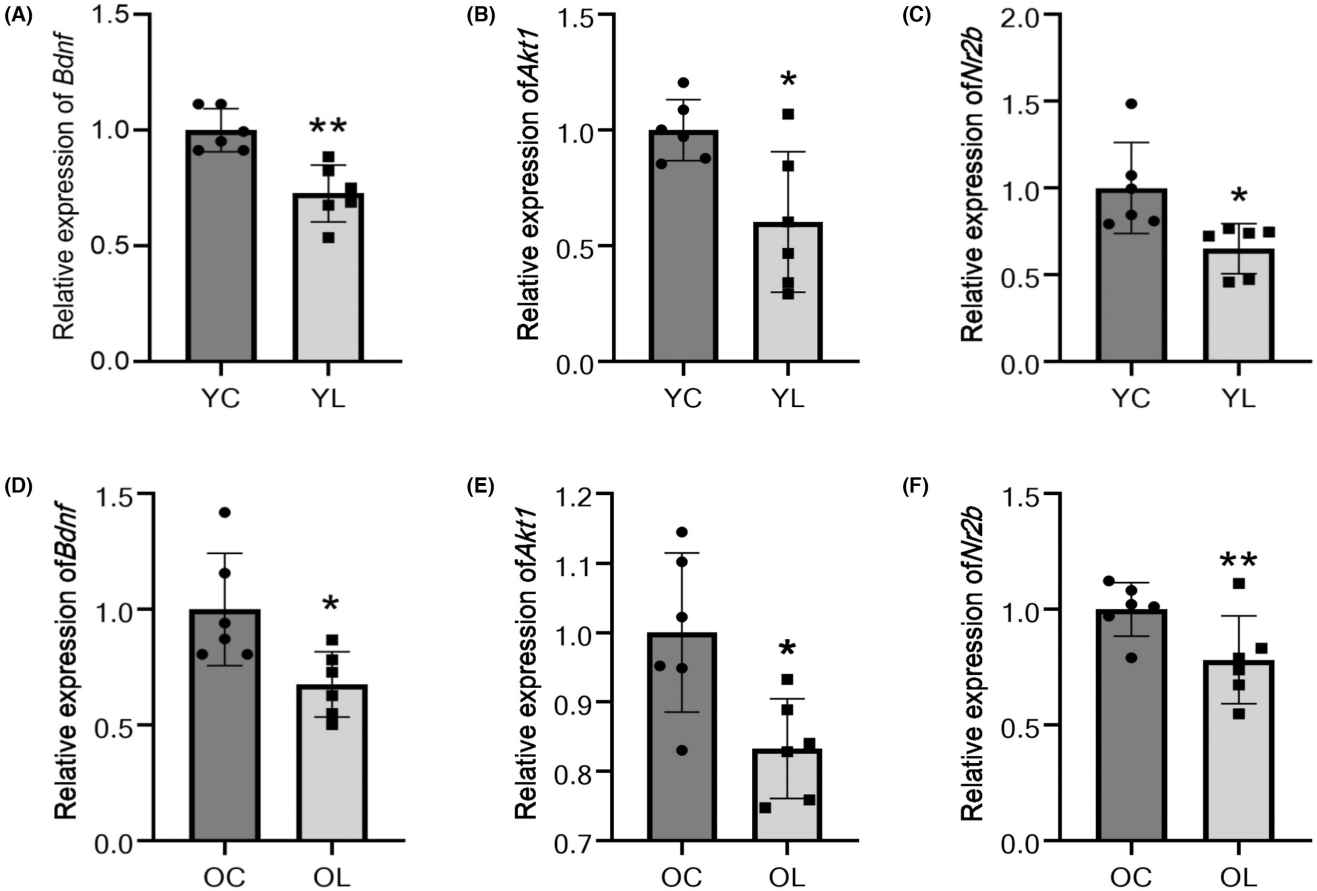
The results of qPCR. (A) The expression of *Bdnf* in the hippocampus was significantly lower in rats of the YL group than in those of the YC group (*p* < 0.01). (B) The expression of *Akt1* was lower in the YL group than in the YC group (*p* < 0.05). (C) The expression of *Nr2b* in the YL group was lower than that in the YC group (*p* < 0.05). (D) The expression level of *Bdnf* in the hippocampus was lower in rats of the OL group than in those of the OC group (*p* < 0.05). (E) The expression of *Akt1* in the hippocampus was lower in rats of the OL group than in those of the OC group (*p* < 0.05). (F) The expression of *Nr2b* in the OL group was significantly lower than that in the OC group (*p* < 0.01). Values were means ± SEM of determinations with *n* = 6 for each group. *p < 0.05; **p < 0.01.

The expression of *Bdnf* in hippocampus of rats in YL group was significantly decreased compared with YC group (*p* < 0.01) (Figure 2A), and the expression of *Akt1* and *Nr2b* in YL group was significantly decreased compared with YC group (*p* < 0.05) (Figure 2B,C). The expressions of *Bdnf* and *Akt1* in OL group were decreased compared with OC group (*p* < 0.05) (Figure 2D,E), and the expression of *Nr2b* in OL group was significantly decreased compared with OC group (*p* < 0.01) (Figure 2F).

### 3.4 Decreased protein expression in synaptic‐plastic factors

**FIGURE 3:**
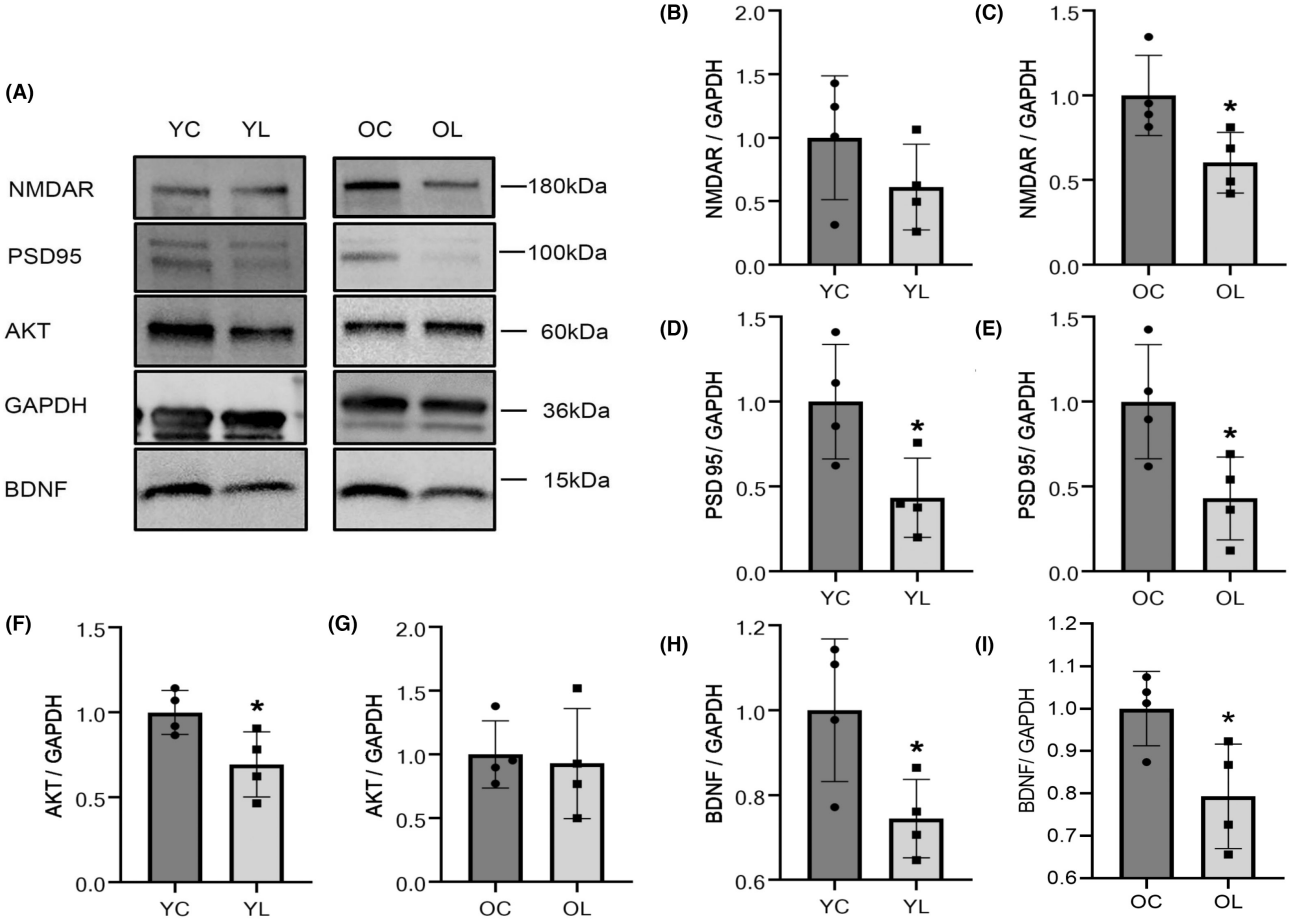
The results of WB. (A) WB strips were shown. (B) The protein expression of NMDAR in the YL group was lower than that in the YC group (*p* > 0.05). (C) The protein expression of NMDAR in the OL group was significantly lower than that in the OC group (*p* < 0.05). (D) The protein expression of PSD95 in the YL group was significantly decreased relative to that in the YC group (*p* < 0.05). (E) The protein expression of PSD95 in the OL group was significantly lower than that in the OC group (*p* < 0.05). (F) The protein expression of AKT in the YL group was decreased compared to YC group (*p* < 0.05). (G) No significant difference in AKT protein expression was seen between the OL and OC groups (*p* > 0.05). (H) The protein expression of BDNF in the YL group was significantly lower than that in the YC group (*p* < 0.05). (I) The protein expression of BDNF in the OL group was significantly lower than that in the OC group (*p* < 0.05). Values were means ± SEM of determinations with *n* = 4 for each group. *p < 0.05.

WB strips are shown in Figure 3A. The protein expressions of NMDAR were decreased in YL compared to YC group, but there was no statistical difference (*p* > 0.05) (Figure 3B). The protein expressions of NMDAR were decreased in OL compared to OC group (*p* < 0.05) (Figure 3C). PSD95 in YL group was significantly decreased compared to YC group (*p* < 0.05), and same trend was shown between OL and OC groups (*p* < 0.05) (Figure 3D,E). AKT in YL group was significantly decreased compared to YC group (*p* < 0.05) (Figure 3F), but there was no significance between OC and OL groups (Figure 3G). BDNF in YL group was significantly decreased compared to YC group (*p* < 0.05), and same trend was shown between OL and OC groups (*p* < 0.05) (Figure 3H,I).

### 3.5 Changes of periodontal tissue‐trigeminal ganglion‐hippocampal pathway after tooth loss

**FIGURE 4:**
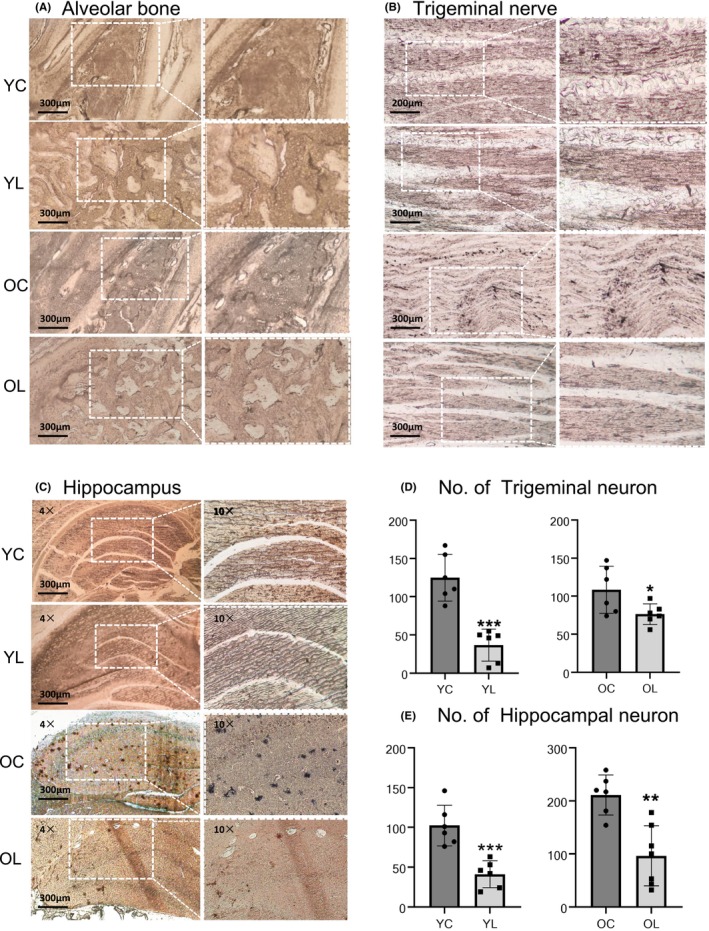
Golgi staining results. (A) The alveolar bone of YL and OL groups at the extraction site had loose structure, and dark brown nerve fibers could be seen rarely in the cancellous bone. In YC and OC groups, the structure of tooth and periodontal membrane were complete with abundant blood vessels, dense dyed nerve fiber bundles and neurons. (B) The structure of trigeminal nerve in YL and OL groups were loose and with less neurons and fibers. (C) Dark brown neurons in the hippocampus were decreased in YL and OL groups. The neurons in hippocampus of YL group were mainly distributed in pyramidal cell layer, with a smaller volume. The volume of neurons in OL group was smaller, with less protrusion and a simpler morphology. (D) The number of dyed neurons in the trigeminal nerve fiber in YL group was significantly less than YC group (*p* < 0.001). While dyed neurons in OL group were less than OC group (*p* < 0.05). (E) The number of hippocampal neuron in YL group was significantly decreased compared to YC group (*p* < 0.001). The number of hippocampal neurons in OL group was significantly decreased compared to OC group (*p* < 0.01). The numbers of rats were 3 for each group. Slices were observed under a 10x light microscope, scale bar = 300 μm. The number of neuron was calculated by Image J, statistical significance was performed using a two‐tailed Student's *t*‐test. *p < 0.05, **p < 0.01, ***p < 0.001.

Dense dyed nerve fibers and neurons in alveolar bone and periodontal membrane were shown in Figure 4. The number of dyed neurons in the trigeminal nerve fiber in YL group was significantly less than YC group (*p* < 0.001), the structure of the trigeminal nerve fiber in OL group was more loose than OC group, and the number of neurons was less than OC group (*p* < 0.05) (Figure 4D). The neurons in hippocampus of YL group were mainly distributed in pyramidal cell layer, with a smaller volume. The number of cells was significantly decreased compared to YC group (*p* < 0.001). The number of hippocampal neurons in OL group was significantly decreased compared to OC group (*p* < 0.01). The volume of neurons in OL group was smaller, with less protrusion and a simpler morphology (Figure 4E).

### 3.6 Location of BDNF/AKT in the hippocampus

**FIGURE 5:**
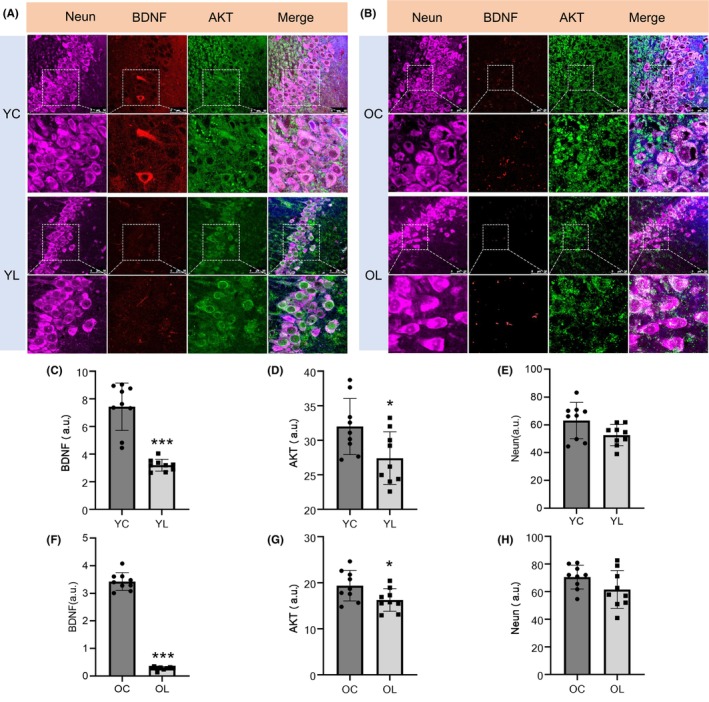
Results of immunofluorescence staining. (A, B) Pyramidal cells: There were fewer pyramidal cell layers in the hippocampal CA1 region of rats in YL and OL groups compared with that in the YC and OC groups. The cellular structure in the YL group was loosely arranged, vacuolated, and showed pleomorphic changes. BDNF: The fluorescence intensity of BDNF in the YL and OL groups was decreased than YC and OC groups (*p* < 0.001). AKT: AKT in YL and OL groups was distributed in the cytoplasm weakly, while AKT in YC and OC groups was widely expressed in the cytoplasm and the inner membrane of the cell. The fluorescence intensity of AKT was significantly decreased in the YL and OL groups than in the YC and OC groups (*p* < 0.05). *n* = 3 for each group. Scale bar = 50 μm. The fluorescence intensity of indicators was calculated by Image J, statistical significance was performed using a two‐tailed Student's *t*‐test. *p < 0.05, **p < 0.01, ***p < 0.001.

As shown in Figure 5, the average fluorescence intensity of BDNF in YL group was significantly lower than YC group (*p* < 0.001). The average fluorescence intensity of BDNF in OL group was significantly lower than OC group (*p* < 0.001). The average fluorescence intensity of AKT in hippocampal CA1 region of YL group was lower than that in YC group (*p* < 0.05). The average fluorescence intensity of AKT in hippocampal CA1 region of OL group was lower than that in OC group (*p* < 0.05). The number of pyramidal cell layers in hippocampal CA1 region of rats in YL group descended compared to YC group, this decrease could also be seen in OL group when compared to OC group. But there was no significance in fluorescence intensity of Neun between YL and YC groups (*p* > 0.05), or between OL and OC groups (*p* > 0.05).

## 4 DISCUSSION

Two age groups were conducted in this study to overview the effects of tooth loss on neuroplasticity in rats. 5‐weeks‐old group was consisting of rats whose nervous systems have not completely developed.^28^ This group was assumed to show more plastic changes in brain while facing external factors,^29^, ^30^ especially when compared to the middle‐age. In this study, the tooth‐extraction was performed referring to former studies, which have been proved to be reliable and mild.^31^, ^32^, ^33^ Gingival division was performed on all maxillary teeth with a pointed probe to reduce excessive surgical trauma and pain,^34^, ^35^ and avoid postoperative cognitive impairment.^36^, ^37^ After extracting bilateral maxillary molars, dental root that might remained should be pulled out, so as to exclude afferent stimulation generated by periodontal membrane.^38^ The surgical trauma was considered to be tolerable. The wounds of the rats were hemostatic when they returned to their cage.

Inadequate intake of nutrients can lead to cognitive impairment, especially in a young age.^39^, ^40^, ^41^, ^42^, ^43^, ^44^, ^45^, ^46^ The body weight of rat was closely monitored after surgery to ensure that the weight recovered and saw stable growth, which lasted for at least 2 weeks before MWM. The weight of rats in YC and OC groups recovered to the preoperative level after 2 and 13 days, respectively. It took more time for middle‐age rats to recover and gaining weight, we assume that with the completion of sexual maturity and jaw development, the difficulty of tooth extraction will be increased,^47^ which may result in more serious extraction damage.

MMW is reported to be a gold standard test to evaluate cognitive level of rat, which avoid interference from food and stress.^48^ In the probing test, the spatial memory ability of YL and OL groups was significantly decreased, which was reflected in the obvious extension of TFPP. Although FPP between groups had no statistically difference, the swimming tracks of rats showed different temporal and spatial characteristics. Most rats that undertook tooth‐extraction tended to explore the pool more randomly, while rats in control groups frequently crossed the platform area. We suspect that the lack of statistical significance is due to the temporal characteristics differed between groups. Rats in YC and OC groups swam in circles around target area in an early stage, but turned to other areas for exploration. Meanwhile, rats in YL and OL groups searched for the platform randomly during the whole 90 seconds. In future experiments, we hope to introduce index to describe the temporal–spatial characteristics of exploration.

Neuroplasticity, which manifested as synaptic plasticity at microscopic level, was reported to be the main mechanism of learning and memorizing.^49^ After an abundant period of bone healing process, we observed decreased numbers of dyed neurons and fibers in alveolar bone, trigeminal nerve and the hippocampus. The result demonstrated a peripheral nerve damage and plastic change of the pathway from tooth extraction. As for the hippocampus, two dyeing methods (viz., Golgi staining and IF) were conducted. CA1 region was the most of interest region, which is responsible for encoding temporal and spatial memories.^50^, ^51^ The pyramidal cell layer of hippocampus is fundamental for memory transformation and storage, and the number of pyramidal cells, particularly in CA1 region, is closely related to memorize and other cognitive functions.^51^ Those cells can be affected by such factors as trauma, pain,^52^ and hypoxia.^9^, ^53^ Thus, the decrease of number, loose arrangement and changes in morphology of pyramidal cells in CA1 region confirmed the damage in hippocampal function.

To further the investigation in memorize function from a molecular level, we examined proteins and pathways associated with synaptic plasticity. PSD95 and NMDAR are proteins consisting PSD,^54^ which conduct information transmission between synapses. As a conservative skeleton protein, the dropping expression in PSD95 can reflect a decrease in the number of synapses.^55^ Also, NMDAR control Ca2+ influx^56^ and activate synaptic function through depolarization. The level of matured BDNF in hippocampus was detected, which has been confirmed to be closely related to synaptic plasticity.^57^, ^58^, ^59^ In this study, qPCR and WB saw same trends in the transcription and expression of PSD95, NMDAR, and BDNF, which confirm synapses related changes in numbers and functions.

Moreover, BDNF can activate the intracellular AKT transduction pathway, which mainly depends on the phosphorylation of AKT and target proteins.^60^ Among others, AKT pathway plays an important role in promoting cell proliferation and apoptosis. AKT is recruited on the cell membrane to trigger cell proliferation, inhibit cell apoptosis, and play a neurotrophic and protective role.^61^, ^62^, ^63^, ^64^ As for the AKT expression, same dropping trend was found in YL and OL groups when compared to YC and OC groups, respectively. But no statistical significance in WB was found between OL group and OC group. Since functional AKT is phosphorylated, we considered that the expression of phosphorylated AKT may influence the results. Besides, IF was introduced to evaluate the AKT expression located in the hippocampus of rats underwent tooth extraction. AKT was weakly expressed in the pyramidal cells from CA1 region, indicated that the activation of the AKT pathway was down‐regulated after tooth loss. Long‐term deregulation of AKT pathway can lead to neuron apoptosis and decreased functional proteins on synaptic membrane, leading to irreversible changes in synaptic function^65^ and memorization.

This study found negative changes of ascending pathways after occlusal support loss, which primarily confirmed our hypothesis on the reduction of masticatory stimulation leading to memory loss. Investments were conducted on the synaptic plasticity to demonstrate changes in neuroplasticity at a microscopic level. However, the stimulation caused by mastication before and after tooth loss still needs to be quantified. In the future studies, electrophysiological studies will be introduced to determine the function of synapses. We will make further investigation in the changes of AKT‐related pathways, among which, PI3K/AKT pathway has been reported to play an important role in synaptic function. Furthermore, future studies will prolong the observation period and observe cognitive changes after dental implantation.

## 5 CONCLUSION

In conclusion, the loss of occlusal support leads to cognitive impairment by exerting a negative influence on the ascending nerve pathway from periodontal tissue to the hippocampus and suppressing synaptic plasticity.

## AUTHOR CONTRIBUTIONS

Xiaoyu Wang and Qian Pang: contributed to the design of the experiments, collection and assembly of all data, data analysis and interpretation, and manuscript writing. Jiangqi Hu: contributed to data analysis and interpretation and writing of the draft. Bin Luo and Yunping Lu: participated in data collection and analysis. Xu Sun and Shixiang Meng: participated in animal behavioral experiments. Qingsong Jiang: conceived the idea, contributed to data interpretation, and finally approved the manuscript. All authors have given approval to the final version of the manuscript.

## CONFLICT OF INTEREST STATEMENT

The authors have no relevant financial or non‐financial interests to disclose.

## Supporting information


Figure S1.

## Data Availability

The authors declare that all the data supporting the findings of this study are available within this article, its supplementary information files, or are available from the corresponding author, who has all relevant data, upon reasonable request.
