# Supplementary figures and images for: Cognitive decline in Sprague–Dawley rats induced by neuroplasticity changes after occlusal support loss
Source: CNS Neurosci Ther. 2024 Jun 19;30(6):e14750. doi: 10.1111/cns.14750 (PMC11187409; doi:10.1111/cns.14750)

OC OL

NMDAR 180kDa  
PSD95 95kDa 112kDa

AKT 56kDa

— 180 kDa  
— 140 kDa

— 100 kDa —  
— 75 kDa —  
— 60 kDa —  
— 45 kDa —  
— 35 kDa —

25 kDa —  
15 kDa —  
10 kDa —

OC OL

GAPDH 36kDa

BDNF 14kDa

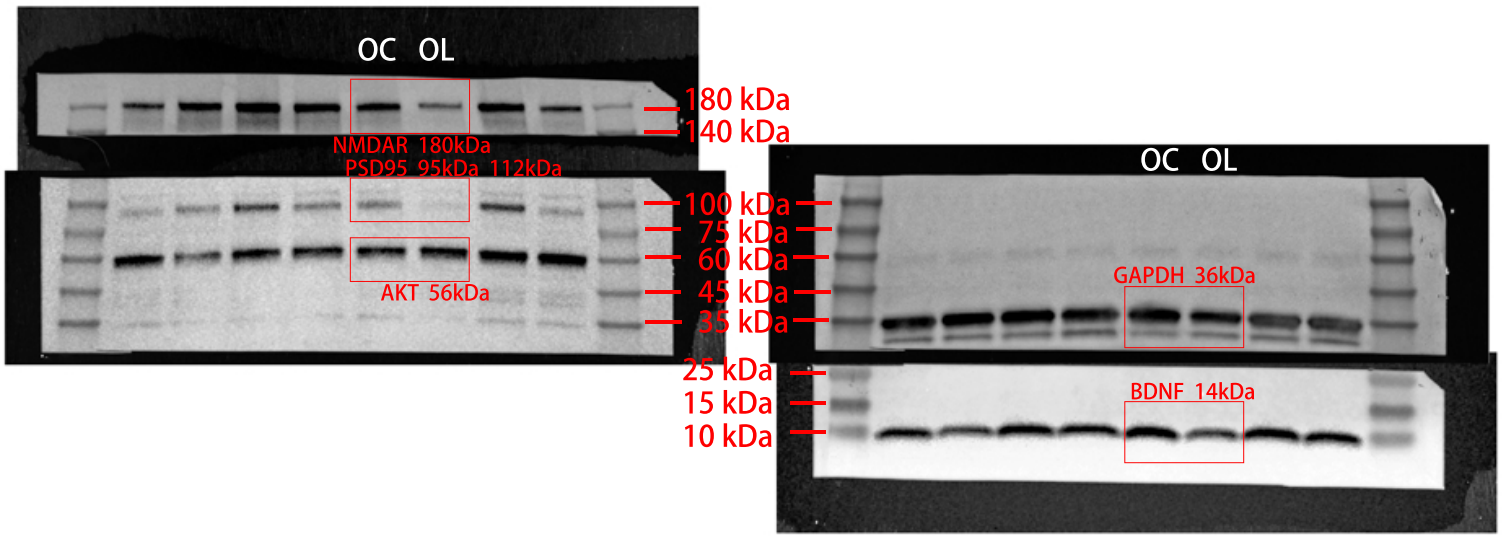

Supplement: Supplementary file 1 — Figure S1. [file CNS-30-e14750-s001.zip › Full unedited blot for Figure 3 (OL and OC groups).pdf]

YC YL

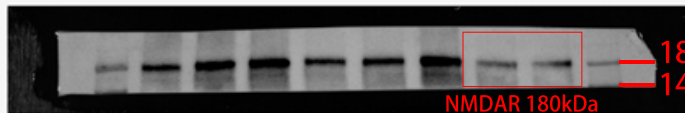

NMDAR 180kDa

PSD 95 112kDa

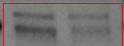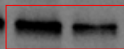

AKT 56kDa

— 180 kDa —  
— 140 kDa —

— 100 kDa —  
— 75 kDa —  
— 60 kDa —  
— 45 kDa —  
— 35 kDa —

25 kDa —  
15 kDa —  
10 kDa —

YC YL

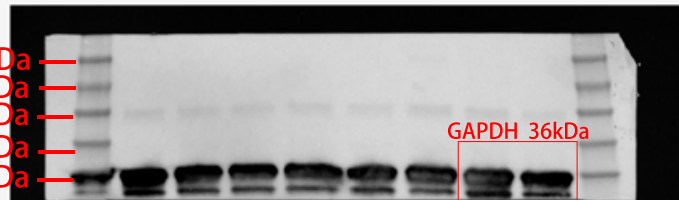

GAPDH 36kDa

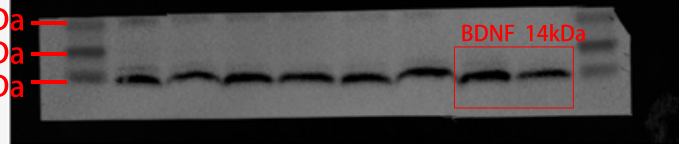

BDNF 14kDa

Supplement: Supplementary file 1 — Figure S1. [file CNS-30-e14750-s001.zip › Full unedited blot for Figure 3 (YL and YC groups).pdf]
